# The Chromatin Signature of Pluripotency: Establishment and Maintenance

**DOI:** 10.1007/s40778-016-0055-3

**Published:** 2016-06-27

**Authors:** Dafne Campigli Di Giammartino, Effie Apostolou

**Affiliations:** Weill Cornell Medicine, Division of Hematology and Medical Oncology, Sandra and Edward Meyer Cancer Center, 413E 69th Street, Belfer research Building, New York, NY 10021 USA

**Keywords:** Pluripotency, Epigenetics, Chromatin architecture, Reprogramming, Stem cells

## Abstract

The revolutionary discovery that somatic cells can be reprogrammed by a defined set transcription factors to induced pluripotent stem cells (iPSCs) changed dramatically the way we perceive cell fate determination. Importantly, iPSCs, similar to embryo-derived stem cells (ESCs), are characterized by a remarkable developmental plasticity and the capacity to self-renew “indefinitely” under appropriate culture conditions, opening new avenues for personalized therapy and disease modeling. Elucidating the molecular mechanisms that maintain, induce, or alter stem cell identity is crucial for a deeper understanding of cell fate determination and potential translational applications. Intense research over the last 10 years exploiting technological advances in epigenomics and genome editing has unraveled many of the mysteries of pluripotent identity enabling novel and efficient ways to manipulate it for biomedical purposes. In this review, we focus on the chromatin and epigenetic characteristics that distinguish stem cells from somatic cells and their dynamic changes during differentiation and reprogramming.

## Introduction

Cell identity is controlled on multiple levels to ensure the maintenance of cell type-defining structural and functional characteristics. The distinct functional properties of ESCs and iPSCs are determined and maintained by a unique combination of transcriptional, epigenetic, and topological features. These features are constantly supervised by a highly interconnected network of ESC-specific TFs, known as master regulators [1–3]. These master regulators of stem cell identity in collaboration with multiple cofactors ensure active transcription of pluripotency-related genes as well as silencing of lineage-specific developmental regulators. Next-generation sequencing approaches mapping TF binding sites, histone modifications, and DNase I hypersensitive sites, revealed a surprising large number of putative regulatory elements with potential activating or repressive function [4•]. These observations clearly indicate that the traditional linear perception of transcriptional regulatory units is insufficient to explain the complexity of gene regulation. In this review, we will focus on the three-dimensional organization of the genome and its involvement in gene control by enabling communication of distal regulatory elements with target genes via chromatin looping. Advances on the mapping of these long-range chromatin interactions, the critical architectural factors, and their importance in cell fate determination will be discussed.

## Functional Genomic Elements of Pluripotency

The pluripotency-specific transcription program is associated with a unique epigenetic landscape. The nature and distribution of histone tail posttranslational modifications as well as DNA methylation patterns largely distinguish embryonic stem cells from somatic cell types. Landmark studies have significantly improved our understanding of the functional significance of specific histone marks or their combinations [5, 6]. Three major chromatin states associated with distinct regulatory elements and transcriptional activities will be briefly discussed here: (i) silent and usually gene-poor heterochromatic regions marked by H3K9me2/3, (ii) poised promoters characterized by co-occurrence of repressive and activating histone modifications (H3K27me3 and H3K4me3, respectively), and (iii) active enhancer types enriched for H3K27ac and H3K4me3, respectively.

It has been long established that heterochromatin is dispersed in ESCs, which are characterized by overall relaxed chromatin structure, [7–9] whereas during differentiation, extensive transcriptionally inactive heterochromatic regions appear. Similarly, reduced overall DNA methylation levels have been described in ESCs, especially in the naive state, which resembles the preimplantation embryonic stem cell stage [10]. However, during development, the total levels increase, since DNA methylation becomes a major repressive mechanism for genes of unrelated lineage [11, 12].

In contrast to somatic cells, the major silencing mechanism in ESCs relies on the recruitment of the polycomb repressive complex (PRC) at the regulatory elements of lineage-specific genes [13, 14] and on the deposition of the repressive H3K27me3 mark. As mentioned above, this histone modification often coexists with the functionally opposing histone mark H3K4me3, thereby establishing an epigenetic state referred to as bivalency [15, 16], which is a defining feature of stem (or progenitor) cell identity. Although these bivalently marked genes are inactive in the pluripotent state, they are poised for rapid activation upon differentiation to the respective lineage by losing the repressive H3K27me3 modification. Therefore, the high plasticity of stem cells is favored by the reversible nature of bivalency as opposed to stable silencing by DNA methylation, which is commonly observed in somatic cells.

Finally, the highly transcribed stem cell-specifying genes are decorated by active histone marks characteristic for promoters or enhancers. It has been proposed that the robust activation of stem cell-specifying genes is controlled by large clusters of regulatory elements composing the so-called superenhancers (SE), which are docking sites for multiple TFs, epigenetic modulators, architectural factors, and basal transcriptional machinery [17]. In addition, recent efforts to globally identify and characterize the functional genomic elements in various cell types revealed that complex networks of distal enhancers can be involved in the tissue-specific regulation of targets genes [4•]. How do these distal regulatory elements communicate with selected target genes? What are the principles of enhancer usage for any given gene? How do master regulators coordinate the expression or repression of their targets genes and which are their critical cofactors? In order to answer these critical questions and gain a better understanding of how gene expression and cell identity are regulated, we need to be reminded that coding and noncoding elements are highly interconnected in the three-dimensional space of the tiny nucleus.

## The Chromatin Shape of Pluripotency

Over the last two decades, it has become increasingly appreciated that the genetic material, which contains all the information for the function and identity of a cell, is not randomly distributed within the nucleus. Technological advances in microscopy and molecular biology have revealed a hierarchical 3D organization of chromatin fibers ranging from chromatin loops between regulatory elements and target genes to larger neighborhoods of frequent interactions and, finally, subnuclear territories [2, 18, 19]. Interestingly, some of the described topological structures, such as the so-called topologically associated domains (TADs), are remarkably conserved across cell types and to some extent even species [20•, 21, 22], suggesting that their formation might be inherent to DNA sequence or to the nature of the chromatin fiber. On the other hand, there are cell-type characteristic architectural features, such as promoter-enhancer looping, which appear to actively regulate cell-type-specific gene expression patterns and may, thus, be crucial for cell fate determination.

**Fig. 1:**
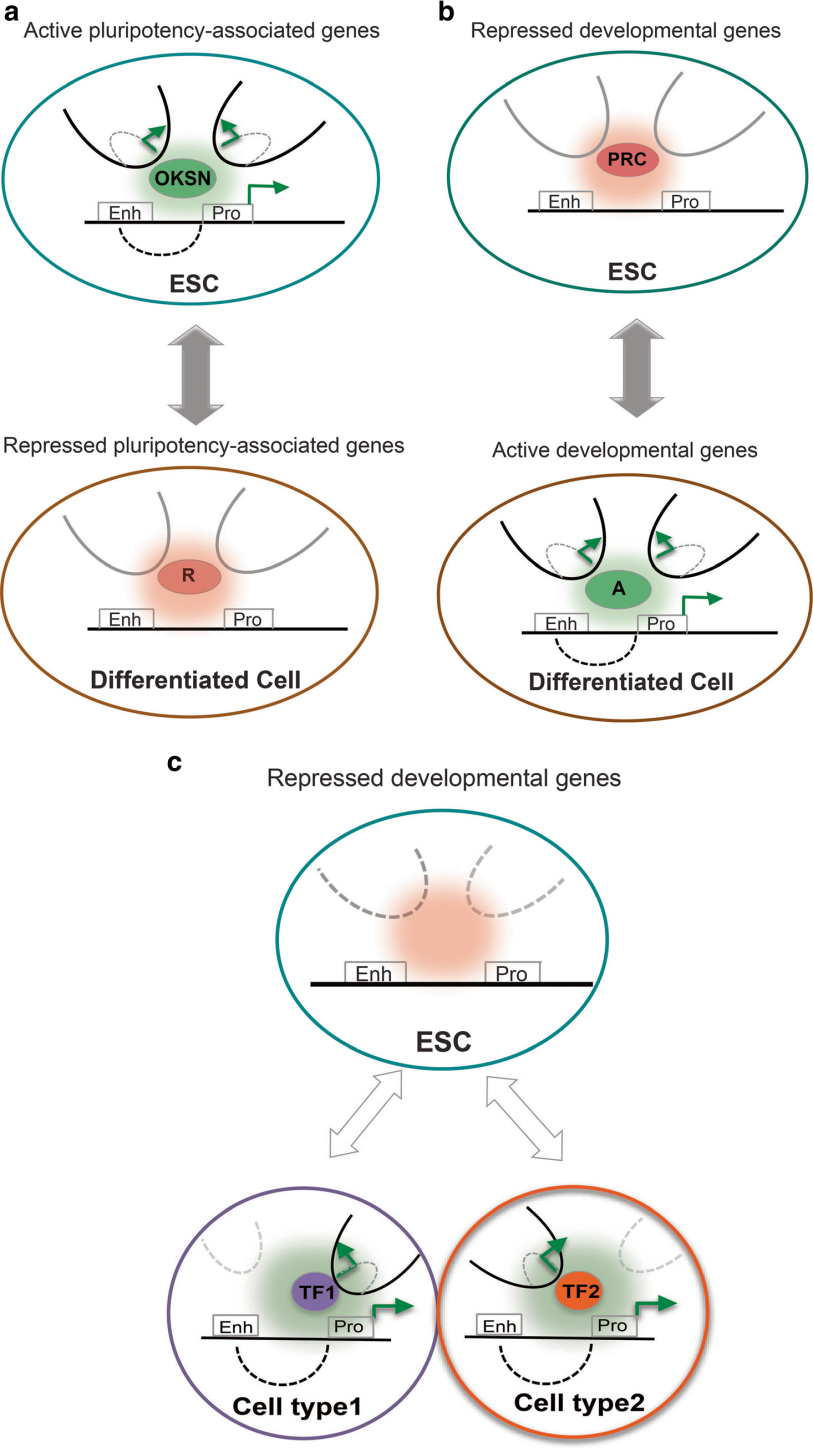
**a** Chromatin interactions among active genes and regulatory elements in pluripotent cells is enriched in OKSN binding. Upon differentiation, the pluripotent-specific loops between enhancer and promoters are disrupted and the respective genes are silenced. **b** Developmental genes in stem cells are silenced through chromatin looping mediated by the repressive polycomb complex. Upon differentiation, the polycomb complex is expected to dissociate and an activator protein will induce the enhancer-promoter looping that leads to gene activation. **c** In this model, inactive lineage-specific genes in stem cells are engaged in promiscuous and dynamic contacts. During lineage specification, the newly activated genes will form contacts mediated by cell-type-specific transcription factors. *OKSN* Oct4, Klf4, Sox2, Nanog, *TF* transcription factor, *PRC* polycomb repressive complex, *Enh* enhancer, *Pro* promoter, *R* repressor, *A* activator

Under the light of chromatin topology, the differentiation process could be envisioned as a reorganization of 3D genomic architecture relative to subnuclear compartments, such as nuclear lamina, or relative to other genes and regulatory elements. During differentiation, pluripotency-related genes have been shown to relocate closer to the “repressive” nuclear periphery and/or toward the interior of their chromosome territory in contrast with lineage-specific genes which follow opposite directions, while they become activated [19, 23•]. An increasing number of chromatin conformation studies in pluripotent, progenitor, and differentiated cell lines reported dramatic reorganization of long-range chromatin contacts [24•, 25•, 26•, 27•, 28•, 29]. Global Hi-C analysis not only corroborated the conserved nature of TADs but also revealed a striking reshuffling of active and inactive chromatin compartments during human ESC differentiation [29]. 5C and 4C studies around key pluripotency or lineage-specific genes identified a large number of long-range chromatin contacts with various degrees of cell-type-specificity, promiscuity, and association to gene expression [24•, 25•, 30]. Although promoter-enhancer loops of critical stem cell genes are robustly formed in ESCs and disrupted in differentiated cells [25•, 27•, 28•, 30, 31•], the connectivity of lineage-specific promoters is less well understood. One study suggested that nonexpressed differentiation-related genes form more random contacts in stem cells [26•], suggesting higher plasticity in chromatin topology. In agreement with this observation, it has been proposed that chromatin topology becomes progressively restrained during the course of differentiation following a topological Waddington landscape [32]. According to this model, stem cells are characterized by a more relaxed configuration, where lineage-specific nonexpressed genes are engaged in promiscuous and dynamic contacts. However, during differentiation, the same genes gradually adopt a more constrained architecture that enables proper gene regulation and prevents regression to the previous flexible state or aberrant expression patterns (Fig. 1).

An alternative model of stem cell chromatin topology could be also envisioned, where the pluripotency-related genes and the lineage-specifying genes are spatially clustered in well-defined active or repressed chromatin hubs. This widely accepted model is supported by a number of key topological studies, such as recent promoter-capture Hi-C experiments, which identified 3D networks of interactions among genes that are coregulated and functionally related [33]. Specifically in ESCs, strong contacts were detected not only among active pluripotency-related genes but also among poised developmental genes, such as the Hox clusters [34•]. Independent studies in ESCs or ESC-derived neuronal cell types described “insulated neighborhoods” of interactions, which are located within TADs and demarcated by cohesin and CTCF-occupied boundaries [24•, 35, 36]. These subdomains contain either active superenhancer-promoter contacts or repressive insulator-promoter contacts. Interestingly, most of these well-defined structures are maintained during differentiation, and thus provide predefined topological units where conformational and transcriptional changes will occur. Interestingly, recent findings in Drosophila embryos support the idea that preformed promoter-enhancer loops of “poised” lineage-specific genes enable their coordinated transcriptional activation during development by release of paused polymerase [37]. Future functional studies in mammals are required to interrogate the biological significance of the spatial proximity among coregulated genes and the underlying mechanisms.

## Architectural Factors of Pluripotency

To better understand the nature of chromatin topology in ESCs and the changes it undergoes during differentiation, it is critical to define the architectural factors involved in shaping, maintaining, or altering long-range chromatin interactions. CTCF is a key genome organizer that was initially described for its insulating properties by mediating looping between promoter and insulator regulatory elements [38]. More recently, CTCF has been proven to be a master architectural factor not only in ESCs [24•, 39] but also in every cell type and species tested, playing a major role in the hierarchical chromatin organization [40]. Specifically, CTCF demarcates the cell-type invariant boundaries of topological associated domains (TADs) as well as the boundaries of subdomains or insulated neighborhoods within TADs [20•, 22, 24•, 35]. The involvement of CTCF in the formation/stabilization of these structures was initially speculated based on the enriched CTCF motif on the boundaries. However, a number of seminal studies that deleted, mutated, or inverted selected CTCF binding sites showed profound effects on TAD integrity, resulting in aberrant loop formation and gene expression patterns [41•, 42•, 43•]. More recently, mathematical modeling of HiC data followed by experimental verification proposed TAD structures are the byproduct of an extrusion process of unknotted chromatin loops by CTCF and cohesin [44]. Ongoing research by multiple laboratories is expected to soon reveal the significance of preferential CTCF binding sites for the formation of TADs, sub-TADs and cell-type-specific chromatin loops, enabling prediction of chromatin topology changes during cell fate transitions or upon genetic and epigenetic alterations around CTCF sites.

In addition to CTCF, mediator and cohesin complexes have also attracted increasing attention for their involvement in chromatin organization over the last 5 years. A pivotal study by Kagey et al. [31•] showed that mediator and cohesin components are critical for the maintenance of pluripotency partly by mediating promoter-enhancer looping of key pluripotency-associated genes. Independent chromosome conformation studies in stem cells and other cell types corroborated the architectural role of mediator and cohesin in a local and genome-wide scale [24•, 25•, 28•, 30]. Interestingly, collaboration of cohesin with CTCF appears to mark cell-type invariant chromatin structures, such as TAD and sub-TAD borders, whereas cohesin together with mediator are usually involved in cell-type characteristic active chromatin loops [24•, 35]. Similarly to cohesin, the Smc2 and Smc4 subunits of the condensin complex have been recently demonstrated to regulate chromatin structure and stem cell identity by colocalizing at high occupancy with architectural protein binding sites in ESCs [45–47]. Of note, both cohesin and condensin complexes have well-established roles in chromosome maintenance during mitosis [48], suggesting the intriguing possibility that other proteins involved in genomic integrity and organization during cell division may also have additional roles in 3D chromatin organization and gene regulation during interphase.

In addition to the classic architectural proteins, there is a growing list of proteins and RNAs with potential role in chromatin looping. Among them, polycomb proteins arise as critical regulators of long-range chromatin interactions. Genomic regions enriched for Polycomb binding and/or its associated repressive histone mark H3K27me3, interact at high frequency in ESCs, and this contact is dependent on the Eed subunit of the PRC2 complex [27•]. In addition, the PRC1 complex functions as a master regulator of ESCs genome architecture by spatially restraining polycomb target genes, such as the Hox clusters and other developmental regulators, in repressive or poised hubs, whose integrity is critical for proper silencing, and thus, stem cell identity [34•]. RNA-mediated gene control is also becoming increasingly relevant for 3D chromatin organization as well as pluripotency. Recent work supports the involvement of ncRNAs in the establishment of promoter-enhancer looping by directly recruiting mediator at these sites [49]. In addition, ncRNAs have been shown to interact with other known architectural factors, such as CTCF and polycomb proteins, contributing to gene regulation via looping [50, 51]. A list of ESC-specific lncRNAs has been recently revealed, arguing for an important role in maintenance of stem cell identity [52]. Although many potential mechanisms have been proposed for ncRNA function [53], one possibility is that ESC-related ncRNAs mediate and stabilize pluripotency-specific chromatin loops in collaboration with other general or cell-type-specific architectural factors. The recently reported interaction of ncRNAs with pluripotency-related TFs, such as Sox2 and Oct4 [51, 54], further supports this scenario, which definitely deserves further study.

Finally, several studies support a potential role of lineage-specifying transcription factors in chromatin loop formation and maintenance. Elegant studies in erythroid cells demonstrated the importance of critical master regulators of hematopoiesis, such as GATA1 and Klf1, not only for promoter enhancer looping around erythropoiesis-related genes [55, 56, 57•] but also for the maintenance of long-range chromatin contacts among several coregulated genes in cis and in trans [58]. Several lines of evidence suggest that ESC-related TFs may play similar architectural roles in pluripotent cells. Chromatin conformation studies showed that regions containing a high density of binding sites for Oct4, Sox2, and Nanog tend to cluster together over large distances and this preference is lost upon lineage commitment [24•, 25•, 26•, 27•, 28•]. Interestingly, the spatial clustering of Sox2-bound enhancers was also captured by high-resolution live imaging in ESC [20•]. In addition, Klf4 protein (highly similar to Klf1) was shown to be important for the maintenance of selected interactions around *Oct4* enhancer [28•] and Nanog protein was sufficient to induce 3D contacts between ectopically introduced Nanog binding sites with endogenous OSN targets elsewhere on the same chromosome [26•]. These results together point to the exciting possibility that pluripotency-associated TFs might actively orchestrate long-range chromatin loops among their target genes. The observations that many pluripotency-related TFs colocalize and/or directly interact with other architectural factors, such as mediator and cohesin [25•, 28•] as well as selected ncRNAs [51, 54], further suggest that all these factors may collaborate to build and preserve the ESC-specific chromatin topology.

## Reestablishment of the Stem Cell-Defining Chromatin Architecture During Reprogramming

Knowing the molecular characteristics and critical regulators of stem cell identity brings us closer to understanding what stemness is and how can be “reestablished” during somatic cell reprogramming to iPS state. Intense research since the discovery of induced pluripotency revealed the dynamic gene expression and epigenetic alterations that somatic cells undergo during reprogramming [59, 60]. However, the reorganization of the chromatin landscape from a somatic to a pluripotent state and the underlying mechanisms remain poorly understood and understudied.

Somatic cell reprogramming is driven by the ectopic expression of a defined set of potent ESC-associated TFs; usually Oct4, Sox2, and Klf4. These factors have been reported to bind nucleosomes in vitro and in vivo through their respective DNA-binding domains, which are able to recognize partial motifs on the nucleosomes [61]. This pioneer activity of selected TFs might be essential for their reprogramming function, enabling binding on the silent inaccessible chromatin of somatic cells and recruitment of important cofactors [62]. Subsequent chromatin and epigenetic remodeling allows the activation of critical stem cell genes and the establishment of a new identity. Whether the pioneer function of reprogramming TFs also includes the recruitment of architectural factors and reorganization of the chromatin contacts remains to be shown. There is only a handful of studies so far that captured the dynamic architectural rearrangements around selected pluripotent or somatic loci during reprogramming. All of them report that the resulting chromatin conformation in iPSCs is highly similar to the one in ESCs, further suggesting that genomic organization is tightly linked to cell identity [25•, 26•, 28•]. The same publications also provide evidence that the observed rearrangements are likely directly driven by OKS and cofactors rather than a consequence of epigenetic and transcriptional changes. The newly established contacts in the course of reprogramming are highly enriched in reprogramming TF binding and especially Klf4 [25•, 29]. Moreover, formation of new chromatin contacts often precedes transcriptional activation of associated genes [28•, 29]. Therefore, one could envision a model where OKSM binding followed by recruitment of architectural cofactors results in spatial clustering of their target genes to coordinate transcriptional activation or repression. In support of such model are the observations that mediator and cohesin directly interact with OKS at early reprogramming stages [25•], and their depletion during reprogramming impairs the generation of iPSCs, partly by interfering with the establishment of pluripotency-specific contacts around the *Nanog* or *Oct4* gene [25•, 30]. Therefore, the induction as well as the maintenance of stem cell-specific interactions in the 3D space seem to be ensured by the direct interplay between these cofactors and stem cell master regulators. Future studies are expected to provide more definite answers on the interplay of TFs with other architectural proteins and ncRNAs and the cause-and-effect relationships between chromatin reorganization and transcriptional changes or epigenetic alterations.

Previous studies reported that iPS often retains epigenetic and transcriptional memory of the starting somatic cell type due to incomplete erasure of the characteristic epigenetic signature. Two recent studies tested whether an equivalent “architectural” memory exists and what the functional consequences would be of incomplete reorganization of chromatin topology to the pluripotent state [63•, 64•]. Both studies demonstrated that the 3D genomic organization of iPSCs derived from different somatic cell types is highly similar to the ESC-specific architecture and strikingly different from the ones of the founder cells. However, Beagan et al. [64•] using high-resolution 5C around selected targets detected local “miswired” interactions that were associated with incomplete transcriptional reprogramming. These results argue for existence of an architectural memory, which though could be erased by exposure of iPSCs in appropriate culture conditions. On the other hand, Krijger et al. [63•] using genome-wide HiC assays in four different somatic cell types and their iPSC derivatives,  detected only subtle de novo topological changes in early-passage iPSCs that correlate with the topology of the respective founder cells. However, the detected topological differences were not associated with any effects neither on the expression of linked genes nor on pluripotency and they likely reflect distinct reprogramming trajectories. The different methodologies, computational analyses, biological material, and reprogramming methods may account for some of the disparities. Future high-resolution conformational studies in different somatic cells undergoing reprogramming will be insightful on how and when stem cell-specific loops are formed and somatic-specific contacts are erased and what their relationship to transcriptional changes.

## Conclusions and Future Perspectives

Function and structure are tightly linked in biology. Elucidating how the same genome instructs fundamentally different cellular programs and phenotypes requires a better understanding of its 3D architecture. Over the last decade, important discoveries have been made, starting to unravel the basic principles of chromatin organization. However, many questions pertaining to the molecular mechanisms orchestrating long-range chromatin interactions and their role in gene regulation and cell identity remain unresolved. How do reprogramming or lineage-specifying TFs promote such dramatic and global chromatin reorganization during reprogramming or differentiation, respectively? Is the formation or disruption of cell-type-specific chromatin contacts a rate-limiting step during cell fate transitions, constituting a new “architectural barrier” in addition to well-described epigenetic barriers? How do multiple regulatory elements with potentially opposing functions control target genes? Addressing these questions will ultimately require high-resolution genomic approaches at the single-cell level and in a time-course manner in order to capture the dynamic and stochastic nature of these events and draw cause-and-effect relationships. In addition, systematic functional genetic screens targeting noncoding regulatory elements involved in looping will identify principles of hierarchical or synergistic enhancer/insulator usage, enabling manipulation of gene expression and cell fate by modulating selected regulatory elements.
